# Serotonin transporter clustering in blood lymphocytes predicts the outcome on anhedonia scores in naïve depressive patients treated with antidepressant medication

**DOI:** 10.1186/s12991-015-0085-8

**Published:** 2015-12-21

**Authors:** Tania Rivera-Baltanas, Roberto Carlos Agis-Balboa, Raquel Romay-Tallon, Lisa E. Kalynchuk, Jose Manuel Olivares, Hector J. Caruncho

**Affiliations:** Instituto de Investigaciones Biomédicas de Vigo (IBIV), Rebullon Psychiatric Hospital, Vigo, Galicia Spain; Division of Pharmacy, College of Pharmacy and Nutrition, University of Saskatchewan, 107 Wiggins Road, Saskatoon, SK S7N 5E5 Canada; Division of Neurology, Department of Medicine, College of Medicine, University of Saskatchewan, Saskatoon, SK Canada

**Keywords:** Biomarker, Depression symptoms, Psychiatric scales, Serotonin, Protein clustering

## Abstract

**Background:**

We have shown that serotonin transporter (SERT) clustering in blood lymphocytes is altered in major depression and correlates with pharmacological therapeutic responses measured with the Hamilton scale. In the present report, we extend these results to the self-assessment anhedonia scale, as anhedonia is a cardinal symptom of major depression that is difficult to treat with first-line antidepressants.

**Methods:**

We collected blood samples from 38 untreated depression patients at the time of enrolment and 8 weeks after pharmacological treatment. We used the self-assessment anhedonia scale to evaluate anhedonia symptoms before and after treatment. We also used quantitative immunocytochemistry to measure SERT clusters in blood lymphocytes.

**Results:**

Evaluation of the distribution of SERT clusters size in the plasma membrane of lymphocytes identified two subpopulations of naive depression patients: Depression I (D-I) and Depression II (D-II). While naïve D-I and D-II patients initially showed similar anhedonia scores, D-II patients showed a good response in anhedonia symptoms after 8 weeks of psychopharmacological treatment, whereas D-I patients failed to show any improvement. Psychopharmacological treatment also induced an increase in the number of SERT clusters in lymphocytes in the D-II group, and this increase correlated with the improvement in anhedonia symptoms.

**Conclusions:**

SERT clustering in peripheral lymphocytes can be used to identify patient response to antidepressant therapy as ascertained by anhedonia scores.

## Background

Major depression is a severe psychiatric disorder characterized by depressed mood, diminished interest or pleasure in most daily activities (anhedonia), fatigue, psychomotor agitation, and suicidal behavior [1, 2]. Major depressive disorder also increases the risk of suicidal ideation, attempted suicide and death by completed suicide. The World Health Organization has further reported that suicide attempts are up to 20 times more frequent than completed suicides and that mental health disorders (particularly depression and substance abuse) are associated with more than 90 % of all cases of suicide [3].

Individuals suffering from depression have increased medical comorbidities and decreased social roles [4]. Although numerous antidepressants have been developed, about one-third of patients are non-responders to these medications [5, 6], and recent meta-analyses suggest that up to 2/3 of patients do not reach remission [7–9]. Therefore, there is interest in identifying biomarkers of therapeutic efficacy, which could significantly advance the development of personalized medical treatment for depression patients [10].

In two recent papers, we have shown that the pattern of clustering of two proteins (the serotonin transporter -SERT- and the serotonin 2A receptor -5HT2A-) in the plasma membrane of peripheral lymphocytes can differentiate two subpopulations of naïve depression patients that show a differential response to antidepressant treatment [11, 12]. We ascertained the efficacy of the treatment by evaluating patient scores on the Hamilton depression rating scale (HDRS) [13]. However, the use of this scale has some limitations, because it does not properly evaluate some relevant symptoms in depression, such as anhedonia [14, 15]. Not only is anhedonia a key symptom of depression, but it also seems to be particularly difficult to treat with first-line antidepressant drug therapies [16–18], and its presence may represent a predictor of poor treatment response [19]. In fact, anhedonia is considered to be one of the most promising endophenotypes of depression [reviewed in 20]. One of us has proposed that the concept of anhedonia could describe a final common pathway within which different phenomena converge (i.e., the so-called anhedonia of schizophrenia would be a different phenomenon than the “true anhedonia” characteristic of depression) [15]. From this idea, a novel anhedonia rating scale called the self-assessment anhedonia scale (SAAS) was developed [21].

In this report, we evaluate alterations in the expression of SERT protein clustering in lymphocytes with respect to the scores in the SAAS anhedonia scale in a population of initially drug naïve depression patients (to avoid any misunderstanding, from here on we use the term depression Total, DT, to refer to the total sample of naïve patients enrolled in the study (*n* = 38), and Depression I (D-I) and Depression II (D-II) to refer to the two subpopulations) [11]. We hypothesized that the pattern of membrane protein clustering in blood lymphocytes will differentiate between naïve depression patients who respond to antidepressant treatment and naïve depression patients who do not respond to antidepressant treatment, as measured by changes in SAAS scores before and after treatment. This study will allow us to define SERT clustering in lymphocytes as a promising candidate biomarker of specific anhedonia symptoms and of their response to antidepressant treatment.

## Methods

### Subjects

This study used the same group of participants [major depression patients (*n* = 38)] who were involved in our two recent studies on membrane protein clustering and HDRS scores in major depression patients [11, 12] (see Table 1). This study is in compliance with the Code of Ethics of the World Medical Association (Declaration of Helsinki), and was approved by the Rebullon Hospital ethics committee, which required the signing of a written informed consent from all participants. The inclusion criteria for the depression group required (a) be at least 18-year old; (b) meet DSM-IV-TR criteria for major depression; (c) have a minimum score of 16 points on the HDRS; (d) present with no other major psychiatric disorder or somatic illness; and (e) be naïve for antidepressant treatment at the beginning of the study. Not being a clinical trial, the psychiatrists treated the patients with the antidepressant medication that they deemed necessary, although most patients received an antidepressant and an anxiolytic (for details, please see Reference [11]).

**Table 1:** Sociodemographic characterization of the depression group

	Depression
Number	38
Age	42.25 ± 2.42
Gender	
Men	15 (39 %)
Women	23 (61 %)
Education	
Primary	20 (52.6 %)
Secondary	12 (28.8 %)
Graduation	6 (18.6 %)
Socioeconomic status	
High	1 (2 %)
Average	30 (79 %)
Low	7 (19 %)
Residence	
Urban	30 (73 %)
Rural	10 (27 %)
Marital status	
Single	6 (17 %)
Married	20 (52 %)
Separated	6 (15 %)
Divorced	3 (8 %)
Widowed	3 (8 %)

### Scoring of clinical scales

A clinical psychologist (blind to the diagnosis and drugs prescribed) collected the demographic data of patients and clinical scores. The SAAS scale was used to assess the severity of anhedonia symptoms in depression before and after 8 weeks of pharmacological treatment. The SAAS is a 27-item scale that scores the “intensity” and “frequency” of specific anhedonia symptoms, as well as the “changes” in those symptoms as perceived by the patient. It also discriminates between items related to “physical,” “intellectual,” or “social” enjoyment [22]. After the patients completed the scales, the clinical psychologist calculated the overall score and the scores on the “intensity,” “frequency,” “change,” “physical,” “intellectual,” and “social” subscales.

We have used the SAAS scale instead of other widely used scales such as the Physical (PAS) and Social Anhedonia (SAS) Scale [21] or the Snaith-Hamilton pleasure scale (SHAPS) [24, 25] because anhedonia rating scales published to date fail to distinguish between the different subtypes of anhedonia [26]. The validity and reliability of these scales have not been adequately established in MDD patients [27–29], and there is a marked overlap between schizophrenic patients and controls on PAS scores [30]. In addition, all these scales unanimously emphasize the experience of pleasure in response to positive stimuli, with little or no attention to diminished drive or motivation, not being capable of measuring the perception of change in the hedonic capacity of the patient [22]. We identified response as a significant improvement in SAAS scale upon pharmacological treatment, if was accompanied of a 50 % decrease in scores in HDRS as evaluated in our previous study using the same patients [11].

### Drawing of blood samples and isolation of lymphocytes

Blood samples were drawn by a trained nurse both at baseline and 8 weeks after pharmacological treatment. Briefly, blood samples were drawn with a BD Vacutainer glass whole blood tube, containing 1.5 ml ADC solution [A-trisodium citrate (22 g/l), citric acid (8 g/l), and dextrose (24.5 g/l)]. We collected three tubes of blood from each patient. Lymphocytes were isolated after centrifugation in Ficoll-Paque Plus, and subsequently washed and re-centrifuged to remove contaminating platelets (for details, see Rivera-Baltanas et al. 2010). Lymphocyte pellets were fixed for one minute in a solution of 1 % paraformaldehyde in phosphate buffer, and maintained for up to 10 days in the fridge (4 °C) before processing for SERT immunocytochemistry.

### Immunocytochemistry

Immunolabeling of the serotonin transporter (SERT) was done by successive centrifugation and re-suspension steps as explained by Rivera-Baltanas et al. [23]. After washing in PBS, lymphocytes were incubated with a solution of human IgG (6:100) in 1 % BSA in PBS to block membrane immunoglobulins. Thereafter, samples were incubated overnight at 4 °C with a solution containing a rabbit anti human SERT antibody (Millipore, AB9322) diluted 1:100 in 1 % BSA in PBS. We have previously characterized this antibody by Western blot [11]. Afterward, the samples were washed in PBS and incubated in the dark at RT for 1 h with a goat anti-rabbit antibody conjugated with Alexa Fluor 488 (Molecular Probes, A11008) diluted 1:200 in 1 % BSA in PBS. After immunolabeling, samples were collected onto Superfrost (+) microscope slides (Fisher Scientific) and mounted with Moviol anti-fading medium (Calbiochem) before being cover-slipped. Samples were maintained in the dark at −20 °C until observation in a confocal microscope.

### Imaging

**Fig. 1:**
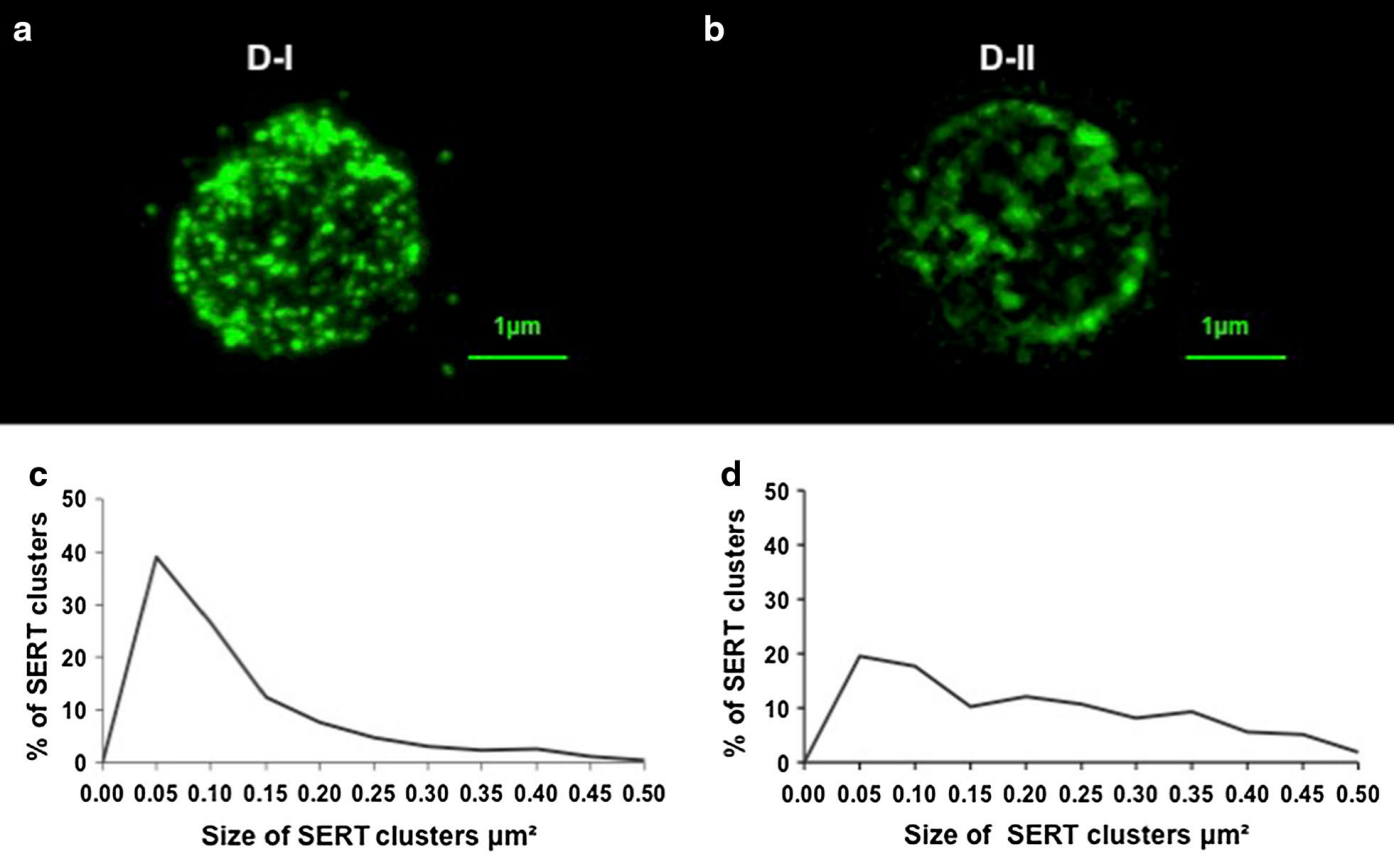
Serotonin transporter (SERT) immunostaining in blood peripheral lymphocytes. SERT labeling is evidenced as immunofluorescent clusters on the plasma membrane of lymphocytes from D-I (**a**) and D-II (**b**) depression patients. The two subgroups of naïve depression patients are differentiated by a different distribution of SERT clusters size, with patients in the D-I group having a modal peak of almost 40 % of SERT clusters between 0.05 and 0.10 μm^2^ (**c**), while this peak is much less evident in patients from the D-II group (**d**). For more details, see references [11, 12]

Immunolabeling of SERT was observed on a spectral confocal microscope (Leica TCS-SP2). We obtained images from 100 lymphocytes per sample. The images were analyzed using the imaging software Image J 1.42 (NIH), which allows for automatic counting of the number of SERT clusters per lymphocyte as well as a calculation of the size of each SERT cluster (see Fig. 1).

### Statistical analyses

All statistical analyses were conducted using IBM’s Statistical Package for the Social Sciences (SPSS v. 20). Two-way ANOVAs (with treatment time -before/after- and drug treatment as the two variables) were used to explore statistical differences in SAAS scores upon medication with different classes of drugs (i.e., antidepressants only, antidepressant plus anxiolytic, antidepressant plus mood stabilizer, or antidepressant plus antipsychotic). The difference in scores SAAS associated with the type of treatment is not our main study variable, but we analyzed the effect of type of antidepressant treatment to know if treatment was conditioned the SAAS scores, so we could consider a variable that could be interfering with the interpretation of our results. We used paired samples t-tests to analyze treatment effects on clinical scales or SERT clustering within each depression group, and one-way ANOVAs followed by post hoc Bonferroni tests to examine differences among groups after treatment. Finally, we carried out one-way ANOVAs to evaluate differences in the possible correlation (analyzed by Pearson’s correlation) between improvement in SAAS scores with treatment, and the increase in the number of SERT clusters induced by treatment. The criterion for statistical significance was set at *p* < 0.05 for all analyses. All graphs depict the mean ± SEM.

## Results and discussion

### Demographic characterization of the subjects

The cohort of depression patients analyzed in this study is the same as the cohort we used in two recent reports on membrane protein clustering of SERT and 5-HT2A receptors [11, 12]. Almost two-thirds of the patients were women, reflecting the epidemiological data showing a higher degree of depression incidence in women compared to men (Table 1). Not being a clinical trial, the patients were prescribed the medication that the psychiatrists felt was the best for each case. However, there were no significant differences in the therapeutic outcome depending on the class of drugs prescribed (for details, please see reference [11]).

### Analysis of SERT clusters in lymphocytes from depression patients

Figure 1a, b shows an example of SERT labeling in the plasma membrane of lymphocytes in D-I and D-II depression subgroups. The present study includes the same patients than in our previous study of SERT clusters in depression in relation to alterations in the Hamilton scale [11] where we identified these two subgroups based on differences in the distribution of SERT clusters size (an example of the different SERT clusters size distribution in D-I and D-II patients is shown in Fig. 1c, d).

### Analysis of SAAS scores evidences no differences between D-I and D-II naïve depression patients, but significant improvement upon treatment only in D-II patients

**Fig. 2:**
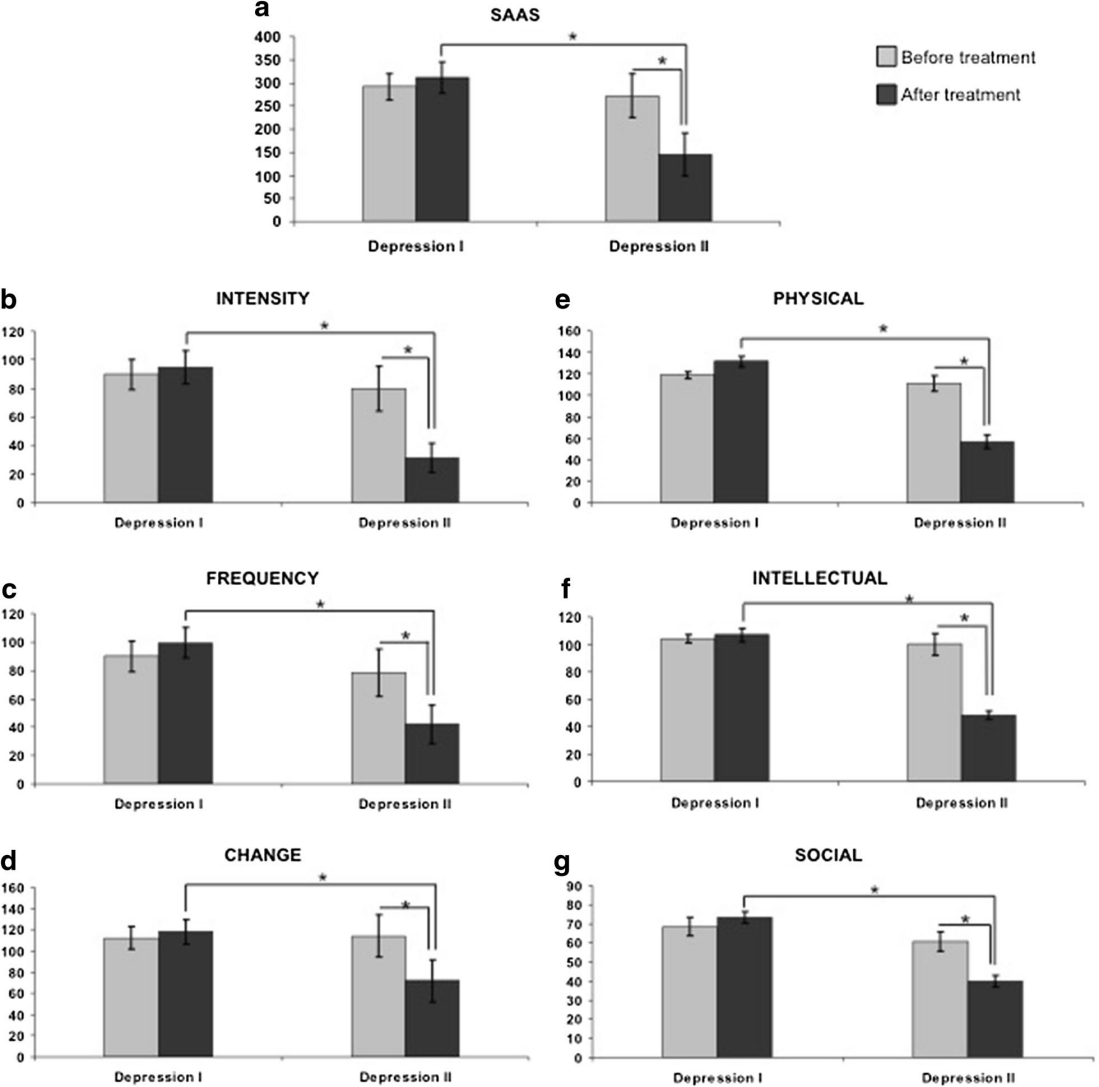
Histograms depicting the variation in the self-assessment anhedonia scale (SAAS) and the different subscales in the D-I and D-II depression groups after psychopharmacological treatment. For details on the SAAS scale, see Ref. [22]

Table 2 shows the SAAS scores and the scores for the different anhedonia subscales (intensity, frequency and change of symptoms, or items related to physical, intellectual, or social anhedonia). There were no statistically significant differences between D-I and D-II naïve patients in any of the scores (before treatment). However, after 8 weeks of pharmacological treatment, there was a significant improvement in anhedonia symptoms within the D-II group only (Tables 2, 3; Fig. 2). Specifically, patients in the D-II group showed a decrease of 47 % in SAAS scores, and also significant decreases in all the subscales (61 % decrease in Intensity, 46 % in Frequency, 37 % in Change, 49 % in physical anhedonia symptoms, 52 % in intellectual symptoms, and 34 % in social symptoms). In fact, the D-I group patients not only failed to show improvement in anhedonia symptoms after treatment, but their overall anhedonia scores after treatment also showed a tendency to be higher than before treatment (Table 2). In addition, although the SAAS scores in the D-I and D-II groups in naïve patients were similar, there were significant group differences after treatment, with the D-II group showing lower levels of anhedonia as measured by SAAS scores (Table 2; Fig. 2). SAAS scores after treatment were 53 % lower in the D-II than in the D-I group, and they also were lower for the subscales (66 % lower in Intensity, 58 % in Frequency, 39 % in Change, 66 % in physical symptoms, 55 % in intellectual symptoms, and 41 % in social symptoms).

**Table 2:** Analysis of SAAS scores before and after treatment

	Before treatment			After treatment		
	DT	D-I	D-II	DT	D-I	D-II
SAAS	287.37 ± 24.7	291.85 ± 2946	272.25 ± 47.58	285.40 ± 31.56	312.26 ± 33.74	145.40 ± 45.98
Intensity	87.46 ± 8.85	89.70 ± 10.6	79.87 ± 15.6	84.71 ± 10.44	94.78 ± 11.58	31.40 ± 10.33
Frequency	87.34 ± 9.1	90.01 ± 10.8	78.35 ± 16.5	89.07 ± 9.88	99.44 ± 10.57	41.98 ± 13.5
Change	112.57 ± 9.2	112.14 ± 10.81	114.03 ± 20.11	111.6 ± 10.67	118.04 ± 11.45	72.02 ± 20.38
Physical	114.50 ± 10.54	116.50 ± 12.59	111.00 ± 19.42	118 ± 13.03	130.28 ± 13.90	57 ± 21.58
Intellectual	102.17 ± 9.96	102.81 ± 12.17	100.13 ± 16.36	98.02 ± 12.52	107.2 ± 13.98	48.5 ± 1347
Social	67.74 ± 7.23	68.69 ± 8.49	64.64 ± 14.51	68.78 ± 7.02	72.21 ± 7.79	42.91 ± 22.21

*DT* depression total (*n* = 38), *D*-*I* depression I (*n* = 30), *D*-*II* depression II (*n* = 8)

**Table 3:** Analysis of SAAS scores before and after treatment

	Depression total	Depression I	Depression II
SAAS	t(37) = 1.226; p > 236	t(29) = −0.210: p > .836	t(7) = 3.054; p < .002
Intensity	t(37) = 1.176; p > 211	t(29) = −0.036: p > .972	t(7) = 3.113; p < .002
Frequency	t(37) = 1.055; p > .284	t(29) = −0.275: p > .786	t(7) = 2.449; p < .04
Change	t(37) = 1.062; p > .296	t(29) = −0.279: p > 783	t(7) = 2.543; p < .04
Physical	t(37) = 1.009; p > 320	t(29) = −0.437: p > 665	t(7) = 2.91; p < .02
Intellectual	t(37) = 1.343; p > 189	t(29) = −0.059: p > 953	t(7) = 3.44; p < .01
Social	t(37) = 1.116; p > 271	t(29) = −0.037: p > 970	t(7) = 2.38; p < .05

### Improvement in SAAS scores after pharmacological treatment in D-II patients correlates with the increase in the number of SERT clusters in lymphocytes

As shown in Table 4, there is a negative correlation (i.e., a decrease in anhedonia scores correlates with an increase in the number of SERT clusters per lymphocyte) that is observed for SAAS and all the subscales, with the exception of the subscale measuring the change in preferences (one of the subscales where D-II patients show a relatively low improvement, see Table 2).

**Table 4:** Correlation between the treatment effect on SAAS scores and SERT clustering in depression II subgroup

	Number of SERT clusters		
	N subgroup D-II	Pearson correlation	Sig. (2-tailed)
SAAS	8	−0.942*	0.017
Intensity	8	−0.897*	0.039
Frequency	8	−0.916*	0.029
Change	8	−0.725	0.166
Physical	8	−0.904*	0.035
Intellectual	8	−0.911*	0.031
Social	8	−0.924*	0.021

* Correlation is significant at the 0.05 level (2-tailed)

## Discussion

The main finding observed in this study is that upon 8 weeks of pharmacological treatment, patients within the D-II depression subgroup show a good response (evidenced by significant improvement of anhedonia symptoms as scored by the SAAS scale), whereas D-I patients fail to show any improvement in anhedonia symptoms. In addition, we also demonstrate that this effect is observed for all the SAAS subscales, and that the improvement in anhedonia symptoms in the D-II group correlates with the level of increase in the number of SERT clusters within the membrane of peripheral lymphocytes after pharmacological treatment. These results are primarily discussed in the following paragraphs in relation to the possible clinical importance of the application of anhedonia clinical scales and analysis of SERT clustering in lymphocytes to evaluate major depression prognosis and therapeutic efficacy of antidepressant treatment in individual patients.

In two recent reports, we have analyzed the variations in membrane protein clustering in lymphocytes in depression of two serotonergic markers (SERT and the 5-HT2A receptor), and demonstrated the existence of two subpopulations of naïve depression patients D-I and D-II. Lymphocytes from D-I patients have about 40 % of SERT/5-HT2A clusters within the modal peak of 0.05–0.10 μm^2^ in size, whereas in lymphocytes from D-II patients, only about 25 % of SERT/5-HT2A clusters fall into this category [11, 12]. Following this categorization, the naïve depression patients were assigned to the D-I or D-II groups (30 patients were observed as D-I and 8 patients as D-II). These two subpopulations are distinguished by the distribution of receptor clusters size, despite showing similar scores on the HDRS [11, 12]. In the present report, we used a similar approach (as the depression cohort is the same one used in our previous studies) and evidence that scores in the SAAS scale are also similar between D-I and D-II naïve depression groups.

Interestingly, our previous study of SERT clusters in depression demonstrated that although patients in both the D-I and D-II groups showed a significant response after 8 weeks of pharmacological treatment (as measured by the HDRS), the patients in the D-II group showed a better response than those in the D-I group, with ¾ of D-II patients showing remission of symptoms [11]. Assessment of SAAS scores also indicates that D-II group patients show a significant improvement in anhedonia symptoms after pharmacological treatment; however—differently than the observations in HDRS scores—patients in the D-I group showed no significant improvement in anhedonia symptoms, and in fact they showed a tendency to an increase in SAAS scores (see Table 2). This implies that analysis of membrane protein clustering of SERT and/or 5-HT2A (as the D-I and D-II depression subpopulations have been equally demonstrated by employing any of those markers as we previously have shown [11, 12]), identifies two subpopulations of naïve depression patients, one of which (the D-II group) shows a good improvement in anhedonia symptoms upon pharmacological treatment, while the other group (i.e., D-I) fails to show any response. One should, therefore, consider the possibility of using the analysis of serotonergic membrane protein clustering in lymphocytes as a way to identify naïve depressive patients that will show a good or bad response to conventional antidepressant medication as assessed by the measurement of anhedonia symptoms.

Improvement of anhedonia symptoms in D-II patients is observed not only in the general SAAS scores, but also in all the subscales, albeit the highest improvement is found in the score on intensity of symptoms, and in the intellectual and physical anhedonia symptoms, whereas for D-I patients, they fail to improve not only in general SAAS scores but also in all the subscales. These findings reinforce our previous suggestion that the pattern of membrane protein clustering could be used to identify patients who will show a good or bad therapeutic response to antidepressant medication.

When analyzing the alterations in SERT clustering in lymphocytes after pharmacological treatment, we found a 27 % increase in the number of SERT clusters per lymphocyte only in D-II patients. Therefore, we wanted to analyze if the improvement in SAAS scores in D-II patients correlates with an increase in SERT numbers. As shown in Table 4, there is a negative correlation (i.e., a decrease in anhedonia scores correlates with an increase in the number of SERT clusters per lymphocyte) that is observed for SAAS and all the subscales, with the exception of the subscale measuring the change in preferences (one of the subscales where D-II patients show a relatively low improvement, see Table 2). We do not know the possible consequences for lymphocytes physiology of an increase in the number of SERT clusters, but it is of interest to note that an increase in interleukin 2, a cytokine primarily released by T lymphocytes in periphery (and also expressed in CNS) is known to have an effect on anhedonia behavior [31].

It will be necessary to perform additional studies to understand the implications of alterations in membrane protein clustering in lymphocytes on the secretion of cytokines, and to perform a clinical trial to properly confirm the findings of this report, but one could surmise the possibility that patients in the D-I group (that would respond poorly to conventional antidepressant treatment as ascertained by measurement of anhedonia symptoms) could be prescribed unconventional antidepressants like bupropion [32] or agomelatine [33, 34] from the start. In addition, it should be logical to study the possible use of the increase in SERT clusters on lymphocyte cell membranes as a possible surrogate marker of antidepressant efficacy, during the development of new antidepressants.

### Limitations

The small sample size may have weakened the power of statistical analyses. This is not a controlled clinical trial, each patient received treatment following clinical practice, mainly the combination of an antidepressant with an anxiolytic treatment. The present study should be followed by a proper double-blind controlled clinical trial to validate the data.

The study is based on the principles of a new paradigm and a new scale of anhedonia which has no other comparable instrument as a gold standard.

## Conclusions

We have shown that analysis of membrane protein clustering in peripheral lymphocytes can be used to identify subgroups of naïve depression patients that show a different outcome in anhedonia symptoms after antidepressant treatment. We suggest that analysis of membrane protein clustering could be a promising candidate used as a biomarker of therapeutic efficacy in anhedonia symptoms for patient stratification in clinical trials.
